# A Novel Anti-TRPV6 Antibody and Its Application in Cancer Diagnosis In Vitro

**DOI:** 10.3390/ijms24010419

**Published:** 2022-12-27

**Authors:** Aurélien Haustrate, Adriana Mihalache, Clément Cordier, Pierre Gosset, Natalia Prevarskaya, V’yacheslav Lehen’kyi

**Affiliations:** 1 Laboratory of Cell Physiology, INSERM U1003, Laboratory of Excellence Ion Channels Science and Therapeutics, Department of Biology, Faculty of Science and Technologies, University of Lille, 59650 Villeneuve d’Ascq, France; 2 FONDATION ARC, 9 rue Guy Môquet, 94830 Villejuif, France; 3 Service d’Anatomie et de Cytologie Pathologiques, Groupement des Hôpitaux de l’Institut Catholique de Lille (GHICL), 59000 Lille, France

**Keywords:** antibody, TRPV6 channel, diagnostic, immunoblotting, immunohistochemistry

## Abstract

Though the first discovery of TRPV6 channel expression in various tissues took place in the early 2000s, reliable tools for its protein detection in various cells and tissues are still missing. Here we show the generation and validation of rabbit polyclonal anti-TRPV6 channel antibodies (rb79–82) against four epitopes of 15 amino acids. Among them, only one antibody, rb79, was capable of detecting the full-length glycosylated form of the TRPV6 channel at around 100 kDa. The generated antibody was shown to be suitable for all in vitro applications, such as immunoblotting, immunoprecipitation, immunocytochemistry, immunofluorescence, etc. One of the most important applications is immunohistochemistry using the paraffin-embedded sections from cancer resection specimens. Using prostate cancer resection specimens, we have confirmed the absence of the TRPV6 protein in both healthy and benign hyperplasia, as well as its expression and correlation to the prostate cancer grades. Thus, the generated rabbit polyclonal anti-TRPV6 channel antibody rb79 is suitable for all in vitro diagnostic applications and particularly for the diagnosis in clinics using paraffin-embedded sections from patients suffering from various diseases and disorders involving the TRPV6 channel.

## 1. Introduction

The TRPV6 channel is a member of the superfamily of transient receptor potential (TRP) channels, subfamily vanilloid, member six [1]. Among all TRP channels, TRPV6 is highly Ca^2+^ selective, with P_Ca_/P_Na_ values exceeding 100; such high Ca^2+^ selectivity is unique within the TRP superfamily (in addition to closely related TRPV5) and makes this channel quite distinguishable, especially in Ca^2+^-related intracellular pathways [2]. Indeed, due to its high calcium selectivity over other TRP channels, this channel was shown to participate in the close regulation of calcium homeostasis in the body [3].

TRPV6 acts as the first step of the transcellular pathway, which is involved in many processes such as Ca^2+^ absorption in the intestine and reabsorption in the kidney [4,5]. TRPV6 was shown to be expressed in the intestine and was also present in the distal tubules of the kidney [6]. TRPV6 is also expressed in the placenta, where it appears to play a role in maternal-fetal Ca^2+^ transport; in the uterus, with a potential role in establishing and maintaining pregnancy; and in exocrine organs such as the pancreas, prostate, mammary, salivary, and sweat glands [3,4]. Studies conducted using *trpv6*^−/−^ mice demonstrated that TRPV6 serves as a principle mechanism for apical intestinal Ca^2+^ absorption [7]. The *trpv6*^−/−^ mice exhibit disordered Ca^2+^ homeostasis, including defective intestinal Ca^2+^ absorption, increased urinary Ca^2+^ excretion, deficient weight gain, and reduced fertility, suggesting the pivotal role in calcium homeostasis in tissues where this channel is expressed.

Though no distinct isoform for this protein is known so far, some alternative translation of this protein has already been reported [8] and may take place in some diseases like cancer, as it was shown previously for the other TRP channel, TRPM8 [9].

It has already been demonstrated that the overexpression of the TRPV6 calcium channel is a common event in cancers of epithelial origin. TRPV6 appears to be up-regulated in tissue samples from ovary, prostate, breast, thyroid, colon, and pancreatic tumors [10]. Despite the discovery of its crucial role in cancer cell proliferation and survival in vitro, no reliable tool to detect the TRPV6 channel has been reported so far to be used in diagnostic applications in vitro.

Among them, the most important applications are the use of the TRPV6 channel as a diagnostic/prognostic marker. Intriguingly, one of the outstanding examples is prostate cancer (PCa) where TRPV6 is absent in healthy prostate and it becomes expressed in prostate adenocarcinoma and TRPV6 expression correlates significantly with the Gleason score, being significantly expressed in lymph node metastasis of prostate origin [4]. Though TRPV6 was proposed as a prognostic marker for advanced prostate cancer in the early 2000s [11], no reliable tool to detect TRPV6 channels in clinics has been reported or used for diagnostic purposes so far. The aim of this work was to create such a tool and show its potential use in cancer diagnostic applications in vitro.

## 2. Results

### 2.1. Generation of Four Rabbit Polyclonal Antibodies

**Figure 1:**
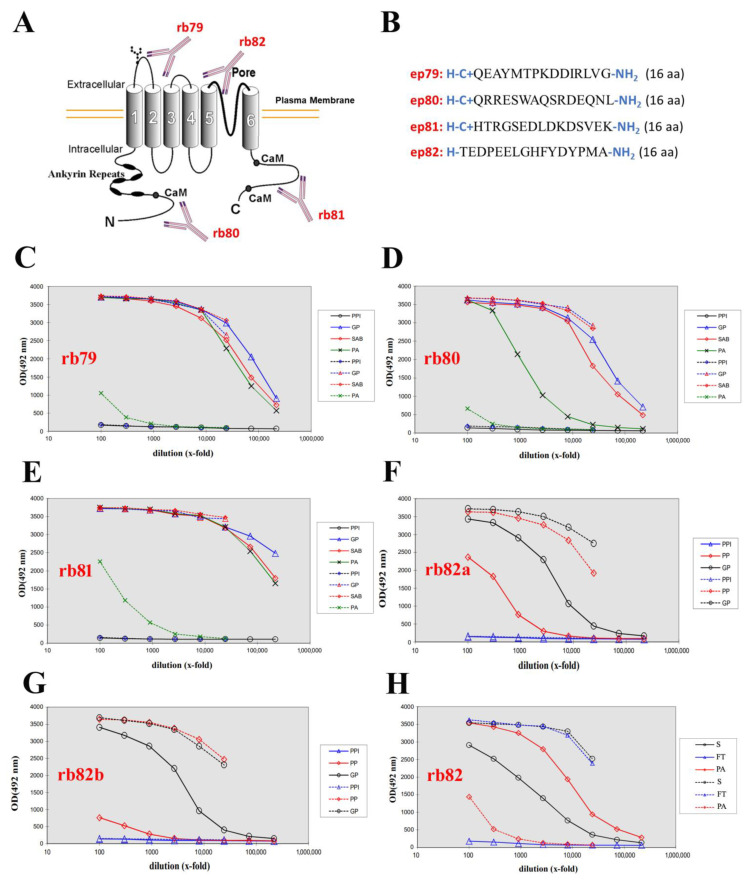
Design and immunoblotting of different rabbit polyclonal anti-TRPV6 channel antibodies. (**A**), The general scheme of the channel and the relative position of the epitopes for the four antibodies, 79–82 (base image of the channel from http://atlasgeneticsoncology.org/, accessed on the 1 May 2009). (**B**), the corresponding epitopes used to generate anti-TRPV6 antibodies, and C—cysteine used to couple with the KLH protein. (**C**–**H**), ELISA analysis using immobilized peptides. PPI—pre-immune serum, GP—large bleed, SAB—final bleed, PA—purified antibodies, PP—small bleed, S—serum, FT—flow through. Straight line—peptide; discontinuous line—carrier. (**C**), the ELISA analysis of the rb79 antibody. (**D**), the ELISA analysis of the rb80 antibody. (**E**), the ELISA analysis of the rb81 antibody. (**F**), the ELISA analysis of the rb82a antibody (rabbit 2358). (**G**), the ELISA analysis of rb82b antibody (rabbit 2359). (**H**), final bleed, and antibody purification from rabbit 2358 followed by ELISA analysis of the rb82 antibody.

The entire TRPV6 protein sequence from UniProt (https://www.uniprot.org/uniprot/Q9H1D0) has been analyzed, and four unique epitopes covering distinct domains, such as the N-terminus, X-loop, pore, and C-terminus, were designed to generate rabbit polyclonal antibodies (Figure 1A,B). Antigens were synthesized and rabbit polyclonal antibodies generated using the AS-SMAF-SINGLE Rabbit—Speedy 28 days program. To monitor the evolution of an immune response, the dilutions of the pre-immune serum (collected just before starting the immunization) and the large bleed (21 days post-immunization for such a program) were tested in parallel. Each sample is then tested against the antigen and both positive and negative controls. As the antigen is a peptide, the peptide and the protein carrier were tested separately. The generated antibodies were named as follows: rb80 for the N-terminus, rb79 for the extracellular loop (X-loop), rb82 for the pore, and rb81 for the C-terminus of the TRPV6 channel. The ELISA analysis for the pre-immune serum (PPI), large bleed (GP), final bleed (SAB), and purified antibodies (PA) of rb79–81 is shown in Figure 1C–E. The same ELISA analysis was performed on both rabbits for the epitope pore of the channel, but only the pre-immune serum (PPI), small bleed (PP), and large bleed (GP) are shown in Figure 1F,G. Due to the complexity and low antigenicity of the pore antigen, the best rabbit (Figure 1F) has been chosen for the long program of 87 days with the additional weekly boosts. The final ELISA analysis of its serum (S), flow-through (FT), and purified antibodies (PA) is shown in Figure 1H. The latter antibody obtained the name rb82.

Thus, four antibodies were generated against human TRPV6 targeting the N-terminus (rb80), the extracellular loop (X-loop) (rb79), the pore region (rb82), and the C-terminus (rb81) and were subjected to further analysis.

### 2.2. The Expression of TRPV6 Protein Revealed with Rabbit Polyclonal Antibodies

**Figure 2:**
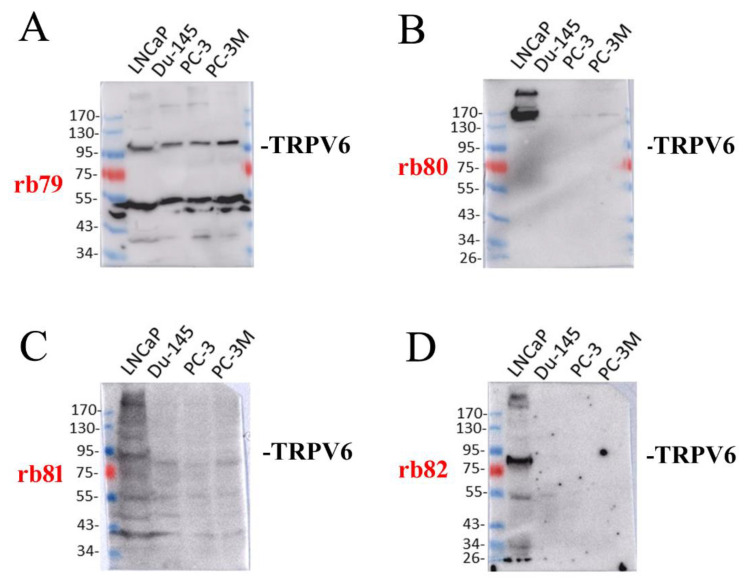
Immunoblotting studies of rabbit polyclonal antibodies rb79–rb82 in different cell lines. The immunoblotting of the total lysates of the LNCaP, DU-145, PC-3, and PC-3M cells in denaturing conditions revealed the presence of the rabbit polyclonal anti-TRPV6 antibodies rb79 (**A**), rb80 (**B**), rb81 (**C**), and rb82 (**D**).

Four generated antibodies were firstly examined using SDS-PAGE in denaturing conditions (Figure 2). In our experiments, we have used a panel of different cancer cell lines known to express TRPV6 [12].

The immunoblotting of total lysates from LNCaP, DU-145, PC-3, and PC-3M cells revealed with rabbit polyclonal anti-TRPV6 antibody rb79 showed an expected size of the glycosylated form of the protein around 95–100 kDa, while the theoretical size of the unglycosylated protein is around 87.3 kDa (Figure 2A). In addition, a band of around 50 kDa was detected, which subsequently turned out to be unspecific despite being present in the ladder lane.

The immunoblotting of the same total lysates revealed with rabbit polyclonal anti-TRPV6 antibody rb80 of prostate cancer cells showed a high molecular band of approximately 160 kDa, which was only detected in LNCaP cells (Figure 2B). In LNCaP cells, immunoblotting with rb81 revealed a multitude of smear-like bands, including one less than 95 kDa (Figure 2C). However, the presence of the smear in many cases raised doubts as to the specificity of this antibody.

Finally, a rb82 antibody showed a staining of one band of 80 kDa exclusively in LNCaP cells, which is below the expected theoretical unglycosylated protein of approximately 87.3 kDa (Figure 2D).

According to the obtained data, all antibodies except for rb79 were discarded since they did not show the expected size of the monomeric protein or the quality of detection.

**Figure 3:**
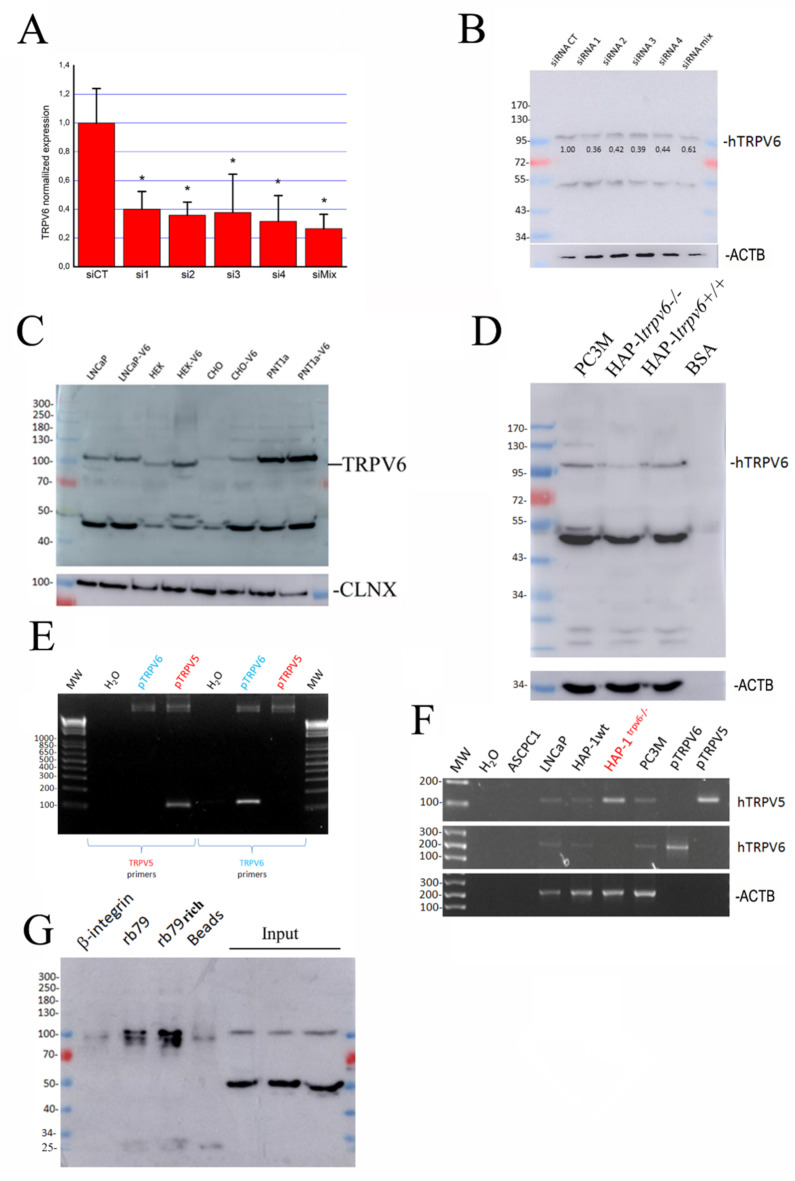
rb79 validation using knockdown, overexpression, and knockout models. (**A**), Quantitative real-time PCR of the TRPV6 channel in the LNCaP cells transfected either with the 40 µM control siRNA (luciferase) or with the 40 nM siRNAs 1–4 against the TRPV6 channel or their mixture as compared to HPRT gene expression (see Table 1 for primers); n = 3, * *p* < 0.05. (**B**), A corresponding immunoblotting of the protein lysates from the siRNA-treated LNCaP cells under the same conditions as in (**A**), and the quantification of the bands as compared to ACTB. (**C**), transfection of the TRPV6 channel using the vEF1ap-5′UTR-TRPV6_CMVp-mCherry vector in LNCaP, HEK, CHO, and PNT1A cell lines and the corresponding immunoblotting. (**D**), TRPV6 protein expression in PC-3M, HAP-1*^trpv6−/−^*, and HAP-1*^trpv6+/+^* cell models versus BSA protein revealed using rb79, as compared to ACTB expression. (**E**), semi-quantitative PCR using TRPV6 and TRPV5 discriminative primers listed in Table 1 on both pTRPV6 and pTRPV5 plasmids. (**F**), semi-quantitative PCR using the same discriminative primers as in (**E**) to detect both TRPV6 and TRPV5 transcripts in various cell lines, including HAP-1*^trpv6−/−^*. Corresponding TRPV5/6 plasmids were used as positive controls, and ACTB expression was used as a housekeeping gene. (**G**), the immunoprecipitation of LNCaP total cell lysates using anti-B-integrin, rb79, rb79-enriched conditions, and beads as a negative control, as well as the input. The blot was revealed using rb79.

To pursue the antibody validation, four siRNAs were used in our studies to carry out a specific knockdown of the TRPV6 channel. The list of the siRNA sequences is indicated in Table 1. They target the first, the seventh, the eleventh, and the thirteenth exons of the mRNA. First, the quantitative real-time PCR of the TRPV6 channel was performed in the LNCaP cells transfected either with the 40 nM control siRNA (luciferase) or with the 40 nM siRNAs 1–4 against the TRPV6 channel (Figure 3A). A mixture of siRNAs has also been used and compared to the housekeeping gene expression, such as HPRT. The efficiency of knockdown at the level of the mRNA was more than 60%, which was reflected in the corresponding immunoblotting of the protein lysates (Figure 3B). The bands have been quantified in comparison to the ACTB (see also Supplementary Figure S2).

As a next step, we used an overexpression system. It should be noted that it is extremely difficult to have a cell system in vitro that does not express the TRPV6 channel since the presence of 2 mM of calcium in almost every medium makes the expression of TRPV6 advantageous for the cell’s survival. Our data show an increase varying from slight to strong in TRPV6 expression (Figure 3C), suggesting that the 100 kDa band is specific to the TRPV6 channel.

Further, we have verified whether in the newly established HAP-1*^trpv6^*^−/−^ cell line the expression of the TRPV6 channel was extinct. Surprisingly, a faint band was still present at the expected size of the TRPV6 channel (Figure 3D). While analyzing the epitope sequence for the rb79 antibody, it was concluded that there were four mismatches between this sequence and the homologous sequence of the closest analog of this channel, TRPV5. Knowing that these channels have strong homology at both peptide and nucleotide sequences (since are the products of the duplication of the same gene in the evolution [13,14]), we first designed a discriminative primer pair allowing the detection of either TRPV6 or TRPV5, and tested them (Figure 3E). Surprisingly, while checking the HAP-1*^trpv6−/−^* cell line model, we have noticed not only a full extinction of the TRPV6 mRNA (obviously by NMDA, nonsense-mediated mRNA decay [15]), but also the overexpression of the TRPV5 channel (Figure 3F). We hypothesized therefore that the overexpression of the TRPV5 channel instead of the TRPV6 channel would allow a slight shift in binding to its close analog sequence of TRPV5. This binding must be really weak and be seen in the immunoblotting experience because of the high sensitivity of the enhanced chemiluminescence reaction used to reveal the signal.

Finally, an immunoprecipitation using the rb79 antibody has been performed to confirm the use of this antibody as well as to see whether one can enrich the quantity of TRPV6. As shown in Figure 3G, the immunoprecipitation of the cell lysates from the LNCaP cell using rb79 (or rb79-enriched protocol) yields two distinct bands that may correspond to both glycosylated and non-glycosylated forms of TRPV6. Interestingly, the unspecific band seen on the blot in Figure 2 at approximately 50 kDa disappears while immune-precipitating cell lysates.

### 2.3. Is There Any Splice Variant of the TRPV6 Protein in LNCaP Cells?

**Figure 4:**
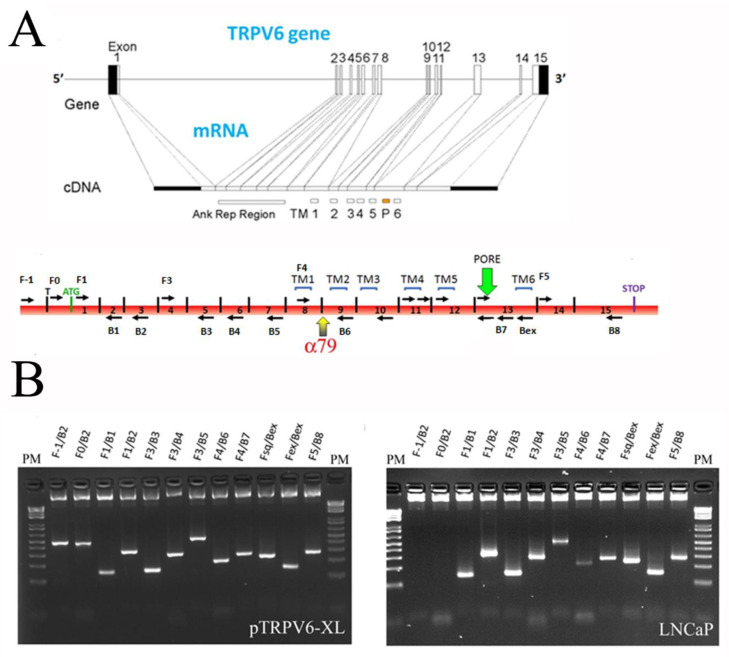
RNA profiling of the mRNA of the TRPV6 channel. (**A**), the general scheme of the TRPV6 gene with the relative position of the introns and exons and the final spliced mRNA of the TRPV6 gene (gene scheme from http://atlasgeneticsoncology.org/, accessed on 1 May 2009), with the position of the primers listed in Table 2 with the expected sizes and targeted exons indicated in the Table 3, the epitope of the antibody rb79, transmembrane, and pore regions. (**B**), RNA profiling using the set of primers and their expected sizes from Table 3 using the vEF1ap-5’UTR-TRPV6_CMVp-mCherry vector alone (left panel) and the total cDNA from the LNCaP cells (right panel).

An RNA-profiling of LNCaP cDNA was performed to determine whether the additional bands seen in SDS-PAGE at around 50 kDa are not alternative forms/splice variants of TRPV6. The *trpv6* gene is situated on the seven chromosome, locus 33–34, and consists of 15 exons (Figure 4A). Our idea was to design in each exon a primer, either forward or a reverse and by crossing them with each other to see whether the exons may be alternatively spliced, if deletions or insertions had occurred, etc. All the primer pairs and the expected sizes of amplicons are indicated in Table 2 and Table 3. We used a plasmid, vEF1ap-5′UTR-TRPV6_CMVp-mCherry, containing the 5′-UTR of TRPV6 as previously reported [8], as a control (Figure 4B, left). All the primer pairs and the combinations thereof were validated. Then, the cDNA from the LNCaP cell line was tested and showed the entire form of the TRPV6 mRNA (Figure 4B, right), suggesting the additional bands revealed by the antibodies might not be specific to TRPV6. In addition, in our cell model, LNCaP cells, the 5′-UTR fragment is not expressed.

### 2.4. Use of the Rabbit Antibody 79 in Immunofluorescence Experiences and FACS

**Figure 5:**
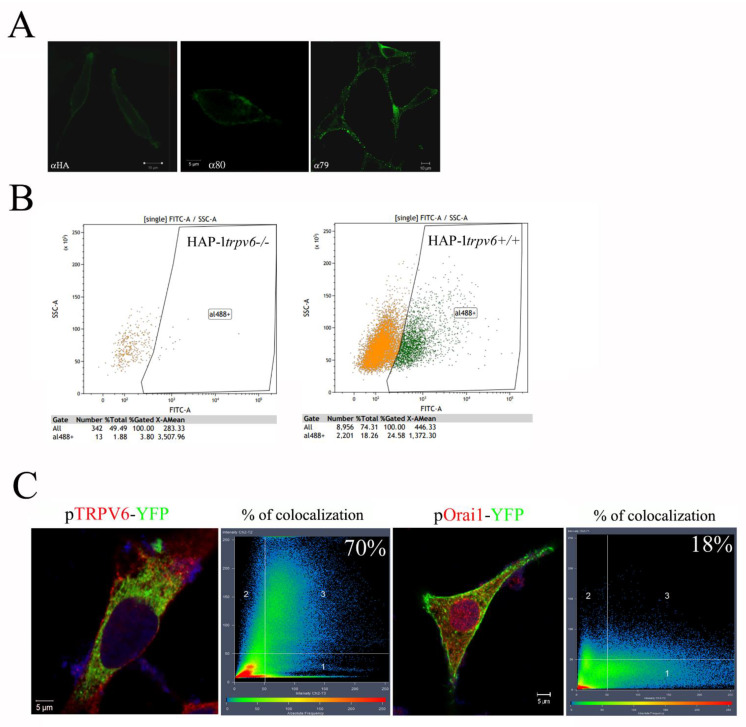
rb79 in plasma membrane TRPV6 staining. (**A**), Immunofluorescence signal (secondary antibody AlexaFluor-488, green) from the 3.5% paraformaldehyde-fixed non-permeabilized LNCaP cells treated with the following primary antibodies: rabbit polyclonal anti-HA tag antibody, rabbit polyclonal anti-TRPV6 antibody rb80 (N-terminus epitope), and rb79 (X-loop). (**B**), TRPV6 surface expression using rb79 in non-permeabilized HAP-1*^trpv6−/−^* versus HAP-1*^trpv6+/+^* cells studied using a FACScan flow cytometer (Becton–Dickinson, San Jose, CA, USA). (**C**), Immunofluorescence signal from the 3.5% paraformaldehyde-fixed, saponin-permeabilized LNCaP cells transfected with either pTRPV6-YFP (left) or pOrai1-YFP (right) plasmids. Both conditions were treated with rb79 followed by the AlexaFluor546 secondary antibody (red). The mean overlapping coefficient between red (TRPV6) and green (YFP) signals is shown as % in the right corner.

Since the rabbit polyclonal anti-TRPV6 antibody rb79 has an extracellular epitope, we initially tried to detect the TRPV6 channel on the outer surface of the plasma membrane using confocal microscopy. Cells were fixed but not permeabilized with the saponin, allowing the exclusive staining of the plasma membrane (Figure 5A). In our experiments, two control antibodies were used: a rabbit polyclonal anti-HA epitope, having the same IgG isotype, and a rabbit polyclonal anti-TRPV6 antibody, rb80, which targets the N-terminus of the TRPV6 channel, which is inside the cell. As it can be seen from Figure 5A, rb79 is capable of staining the plasma membrane TRPV6 channels on the surface of the LNCaP cells.

The additional experiment using FACS has been performed where cells were not permeabilized with Triton X-100. HAP-1^*trpv6−/−*^ cell model has been used as compared to HAP-1^*trpv6+/+*^ cells (Figure 5B). HAP-1^*trpv6+/+*^ cells seem to contain 24.58% TRPV6 positive cells at the plasma membrane versus 3.8% in HAP-1^*trpv6−/−*^.

The established way to test the antibody specificity in immunofluorescence experiments is to transfect the cells with the fusion protein, i.e., the TRPV6 channel coupled to the fluorescent tag, i.e., the pTRPV6-YFP plasmid. Once transfected, the cells are fixed and permeabilized, then incubated with the primary antibody, i.e., rabbit polyclonal anti-TRPV6 antibody rb79, followed by the secondary antibody coupled to Alexa Fluor 546. Further, the analysis of co-localization is done using the microscope-specific software Zeiss Zen. The mean overlapping coefficient between red and green signals (adjusted to the same intensity) is shown as % in the right corner. In our experiment, we used a control plasmid and the other calcium channel, Orai1, fused to the YFP protein. As it can be seen from Figure 5C, the difference is very significant (70% against 18%). This residual discrepancy in the excess of the red signal is explained by the natural presence of the wild type TRPV6 in the LNCaP cell. Thus, the rabbit polyclonal anti-TRPV6 antibody rb79 is suitable for both immunofluorescence and FACS.

### 2.5. Use of the Rabbit Antibody 79 in the Diagnosis and Prognostics in Clinics

**Figure 6:**
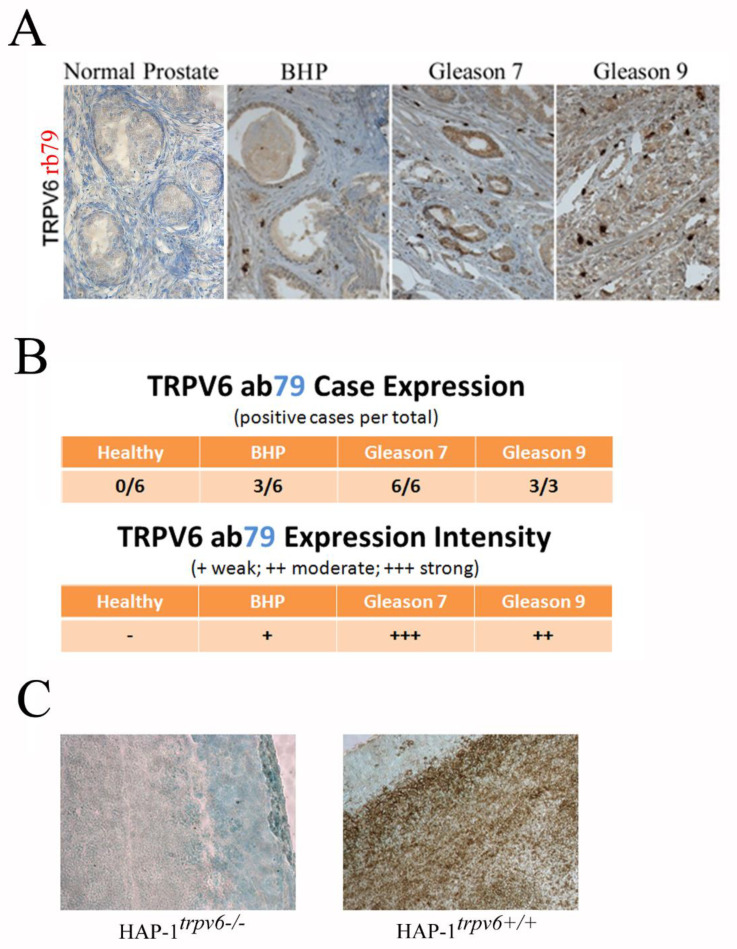
TRPV6 detection in human clinical samples using rb79. (**A**), IHC using rb79 of the human clinical samples from prostate resection specimens in the case of normal prostate (bladder cancer resection specimen), benign hyperplasia of the prostate (BHP), and adenocarcinoma with Gleason scores 7 and 9 (magnification ×200). (**B**), statistical analysis of the number of cases expressed and the intensity of those expressions in clinical samples using RB79 in 21 patient cohorts. (**C**), IHC of tumors slices derived from tumors grafted into nude mice and bearing either HAP-1*^trpv6−/−^* (left) or HAP-1*^trpv6+/+^* (right) cell lines (magnification ×200).

Of four generated antibodies, only rabbit polyclonal anti-TRPV6 antibody rb79 produced a stable and clear staining of prostate cancerous epithelial cells. This antibody has been used to perform IHC using 21 human clinical samples from prostate resection specimens (Figure 6). Six cases of normal prostate (bladder cancer resection specimen), six benign hyperplasia samples of the prostate (BHP), and six adenocarcinomas with the Gleason score of 7 (3 + 4) and three with the score of 9 (4 + 5). The frequency analysis (positive cases per total) and expression intensity (three levels chosen) in clinical samples using rb79 are shown in Figure 6B. Our data confirm the negative expression in healthy prostate and some weak staining in half of the BHP samples which correspond to previously published data [4,12,16,17]. The expression of the TRPV6 channel was also shown to correlate with the expression of the aggressivity markers such as Ki-67, PSA, Chromogranin A, CD31, CD56, Synaptophysin, etc. [12]. It can be concluded that rabbit polyclonal anti-TRPV6 antibody 79 can be used for diagnostic or prognostic purposes.

In addition, rb79 was used to carry out IHC on tumor slices derived from tumors grafted with either HAP-1*^trpv6−/−^* or HAP-1*^trpv6+/+^* cell lines (Figure 6C). As can be seen, the rabbit polyclonal anti-TRPV6 antibody rb79 is not capable of recognizing any TRPV6 channel in HAP-1*^trpv6−/−^*-formed tumors, thereby validating both the knockout model and antibody specificity.

## 3. Discussion

Ion channels are now considered important markers and even hallmarks of cancer [18]. The first mention that the TRPV6 channel appears in some cancers, including PCa, emerged at the beginning of the 2000s. The expression of the TRPV6 channel was mostly studied by two teams, that of Hediger [4] and Flockerzi [17]. Both of them used Northern blot as a technique for TRPV6 detection in tumors. Though the first team detected TRPV6 (previously called CAT1) in benign hyperplasia of the prostate (BHP) [4], the second claimed TRPV6 was not detectable in small sized and confined prostate tumors (grade T1), whereas TRPV6 transcripts were detected in 20% of tumors graded pT2a and pT2b, 79% of pT3a, and more than 90% of pT3b tumors which presented extra prostatic extensions [11] and thus TRPV6 transcripts are not detectable in the normal prostate, in benign, or in high grade prostate intraepithelia neoplasia [12,16]. We have shown that TRPV6 expression may also occur in BHP at a relatively weak level, approximately 50%, and its expression at the protein level increases significantly in adenocarcinomas having the Gleason score of 7 and 9. The exhaustive synopsis of TRPV6 occurrence in various human tumors and the techniques employed to study its expression have been already published [19].

The data previously obtained in prostate cancer allow to suggest the TRPV6 channel as a tumor promoter in the prostate, being expressed de novo it significantly contributes to PCa cells survival as proliferation and apoptosis resistance, promotes the formation of bone metastasis, and potentiates tumorigenesis in vivo. Overall, the increasing evidence suggests that the overexpression of the calcium channel TRPV6 is a common event in cancers of epithelial origin. TRPV6 was observed to be upregulated in tissue samples originating from the ovary, prostate, breast, thyroid, colon, and pancreas [10]. Despite the discovery of its crucial role in cancer cell proliferation and survival in vitro, no reliable tool to detect the TRPV6 channel has been reported so far to be used in diagnostic applications in vitro. In addition, though no isoform for this protein is known so far, some alternative translation of this protein has already been reported [8] and may take place in some diseases like cancer, as it was shown previously for the other TRP channel, TRPM8 [9]. Despite the failure to validate three of the four polyclonal antibodies for TRPV6 detection, a reliable tool to detect TRPV6 expression is of great importance.

In the current work, we show the generation and validation of four rabbit polyclonal anti-TRPV6 channel antibodies (rb79–rb82) against an epitope of 15 amino acids. Among them, only one antibody, rb79, was capable of detecting the full-length glycosylated form of the TRPV6 channel around 100 kDa since this channel contains a strong putative N-glycosylation site as its close analog [20,21]. In our HAP-1*^trpv6−/−^* cell model, the generated antibody is still capable of detecting a slight band of the same size. The precise analysis revealed the four mismatches at the level of the epitope between TRPV5 and TRPV6 proteins. The slightly visible band may be due to the overexpression of its close analogue TRPV5 in the *trpv6*^−/−^ cell model HAP-1. The generated antibody was shown to be suitable for all in vitro applications, such as immunoblotting, immunoprecipitation, immunocytochemistry, immunofluorescence, etc. One of the most important applications we demonstrate is immunohistochemistry using paraffin-embedded sections from cancer resection specimens from 21 patients. Using prostate cancer resection specimens, we have confirmed the absence of the TRPV6 protein in both healthy and benign hyperplasia and its expression and correlation to the prostate cancer grades, as reported previously using Northern blot and PCR [4,12,16,17]. In our previous study, we have also shown that the expression of the TRPV6 channel is associated with the expression of aggressivity markers such as Ki-67, PSA, Chromogranin A, CD31, CD56, Synaptophysin, etc. [12]. These data suggest that having the right tool, such as rabbit polyclonal anti-TRPV6 antibody rb79, will allow it to be used for diagnosis.

In conclusion, the generated rabbit polyclonal anti-TRPV6 channel antibody rb79 is suitable for all in vitro diagnostic applications and particularly for the diagnosis in clinics using paraffin-embedded sections from patients suffering from various diseases and disorders where TRPV6 channels are involved.

## 4. Materials and Methods

### 4.1. Cell Culture

Human PC3M (metastatic cell line issued from PC3 cellules grafted in vivo), PC-3, LNCaP, DU-145, HEK293, and CHO-K1 cell lines were from American Type Culture Collection (ATCC) and were cultured in RPMI (LNCaP, PC3, DU145), DMEM (HEK293), and F12 (CHO) media (Gibco-BRL, Cergy Pontoise, France) supplemented with 10% fetal calf serum, containing kanamycin (100 µg/mL) and L-glutamine (2 mM) where necessary. The HAP-1 cell line is a near-haploid human cell line that was derived from the male chronic myelogenous leukemia (CML) cell line KBM-7 (Essletzbichler, Konopka et al., 2014 [22]) and cultured in IMDM medium (Fisher Scientific, Illkirch, France), supplemented with 10% fetal calf serum and containing kanamycin (100 µg/mL) and l-glutamine (2 mM).

All the cells were cultured at 37 °C in a humidified atmosphere with 5% CO_2_ in the air. The medium was changed three times a week, and cultures were split by treating the cells with 0.25% trypsin (in PBS) for 5 min at 37 °C before reaching confluence. For the experiments, cells were seeded in 6-well plates for PCR and Western blotting. To maintain the *trpv6^−/−^* status of the cells, the antibiotic selection G418 was used at a concentration of 200 µg/mL for the maintenance in culture of the HAP-1*^trpv6−/−^* cells.

### 4.2. SDS-PAGE and Western Blotting

Semiconfluent cells were treated with an ice-cold lysis buffer containing: 10 mM Tris-HCl, pH 7.4, 150 mM NaCl, 10 mM MgCl, 1 mM PMSF, 1% Nonidet P-40, and a protease inhibitor cocktail from Sigma. The lysates were centrifuged at 15,000× *g* at 4 °C for 20 min, mixed with a sample buffer containing (denaturing conditions): 125 mM Tris-HCl pH 6.8, 4% SDS, 5% β-mercaptoethanol, 20% glycerol, 0.01% Bromophenol Blue, and boiled for 5 min at 95 °C. For the non-denaturing conditions, both SDS and β-mercaptoethanol were avoided, and samples were not boiled. Total protein samples (50 µg) were subjected to 8% SDS-PAGE and transferred to a nitrocellulose membrane by semi-dry Western blotting (Bio-Rad Laboratories, Steenvoorde, France). The membrane was blocked in a 5% milk containing TNT buffer (Tris-HCl, pH 7.5, 140 mM NaCl, and 0.05% Tween 20) overnight then probed using specific rabbit polyclonal anti-TRPV6 antibodies (all at 1/500 dilution, incubated overnight while shaking, Table 4), mouse monoclonal anti-β-actin, ACTB, (Lab Vision Co., San Francisco, CA, USA, 1/1000), and mouse monoclonal anti-calnexin, CLNX, (Fisher Scientific, Illkirch, France, 1/1000) antibodies. The bands on the membrane were visualized using an enhanced chemiluminescence method (Pierce Biotechnologies Inc., Rockford, IL, USA). Densitometric analysis was performed using a Bio-Rad image acquisition system (Bio-Rad Laboratories, Heracles, CA, USA).

### 4.3. RT-PCR

RT-PCR experiments were performed as previously described (Lehen’kyi, Flourakis et al., 2007 [19]). Total RNA was isolated using the guanidium thiocyanate-phenol-chloroform extraction procedure. After DNase I (Life Technologies, Saint-Aubin, France) treatment to eliminate genomic DNA, 2 µg of total RNA was reverse transcribed into cDNA at 42 °C using random hexamer primers (Perkin Elmer, Villebon-sur-Yvette, France) and MuLV reverse transcriptase (Perkin Elmer, Villebon-sur-Yvette, France) in a 20 µL final volume, followed by PCR as described below. The PCR primers used to amplify TRPV6 cDNAs as well as TRPV5 and β-actin are specified in Table 1. PCR was performed on the RT-generated cDNA using a GeneAmp PCR System 2400 thermal cycler (Perkin Elmer, Villebon-sur-Yvette, France). To detect different cDNAs, PCR was performed by adding 1 µL of the RT template to a mixture of (final concentrations): 50 mM KCl, 10 mM Tris-HCl (pH 8.3), 2.5 mM MgCl_2_, 200 µM of each dNTP, 600 nM of sense and antisense primers, and 1 U AmpliTaq Gold (Perkin Elmer, Villebon-sur-Yvette, France) in a final volume of 25 µL. DNA amplification conditions included the initial denaturation step of 7 min at 95 °C and 40 cycles of 30 s at 95 °C, 30 s at 60 °C, 30 s at 72 °C, and finally 7 min at 72 °C. The primers used are listed in Table 1. For the RNA profiling, the set of primers used and their expected sizes are listed in the Table 2 and Table 3.

### 4.4. Quantitative Real-Time PCR

The quantitative real-time PCR of TRPV6 and HPRT mRNA transcripts was performed using MESA GREEN qPCR MasterMix Plus for SYBR Assay (Eurogentec, Angers, France) on the Bio-Rad CFX96 Real-Time PCR Detection System. The sequences of primers are indicated in Table 1. The HPRT gene was used as an endogenous control to normalize variations in the RNA extractions, the degree of RNA degradation, and variability in RT efficiency. To quantify the results, the comparative threshold cycle method ∆∆Ct and Biorad CFX Manager Software v2.0 were used.

### 4.5. siRNA Transfection

LNCaP cells were transfected with 40 nM of siRNA against TRPV6 (1–4 or mix) or siLuciferase (Eurogentec, LTD, Seraing, Belgium) using 5 µL of Lipofectamine 3000 transfection reagent (Lipofectamine 3000, Thermo Fisher, Illkirch, France) following the manufacturer’s instructions (see Table 1 for the siRNA sequences). The efficiency of cell transfections with the siRNAs for each particular target has been validated using real-time quantitative PCR and/or Western blotting where appropriate.

### 4.6. Nucleofection

Transfection of various cell lines with different plasmids was carried out using Nucleofector (Amaxa GmbH, Köln, Germany) according to the manufacturer’s instructions. Briefly, 2 µg of the plasmid was transfected into 2 million trypsinized cells, which were then plated onto six-well dishes, 35 mm dishes, or onto the glass coverslips for 48 h.

### 4.7. Confocal Microscopy

LNCaP cells grown on the glass coverslips were washed with PBS and immediately fixed in 4% paraformaldehyde in PBS. PBS-glycine (30 mM) was used to quench the reaction with the subsequent permeabilization with 0.1% Triton X-100. The cells were washed again in PBS and subjected to a conventional immunostaining procedure. Alexa Fluor^®^ 488 goat anti-rabbit IgG (Sigma, Saint-Quentin-Fallavier, France, 1/4000) was used as a secondary antibody for TRPV6 staining. Fluorescence analysis was carried out using a Carl Zeiss Laser Scanning Systems LSM 510 connected to a Zeiss Axiovert 200 M with a 63 × 1.4 numerical aperture oil immersion lens at room temperature. Both channels were excited, collected separately, and then merged using the Carl Zeiss LSM Image Examiner software (3.1.0.99).

### 4.8. Immunoprecipitation

T47D cells grown in the 75 cm^2^ cell culture flask were washed with PBS and immediately lysed with an ice-cold lysis buffer containing: 10 mM Tris-HCl, pH 7.4, 150 mM NaCl, 10 mM MgCl, 1 mM PMSF, 1% Nonidet P-40, and a protease inhibitor cocktail from Sigma. Lysates were incubated with the lysis buffer on the rotating platform for 30 min at +4 °C, then transferred into the 15 mL tube and ultrasonicated for 10 s. The samples were then centrifuged at 15,000× *g* at 4 °C for 20 min, and the total protein concentration was measured. A small amount of the total lysates (50 µg) was kept for the input control, and an equal amount of total protein lysates among different conditions was added to the agarose beads prepared as follows: 50 µL of Agarose-Protein A/G conjugates beads were washed 3 times with the standard PBS pH = 7.4 solution, then the beads were incubated for 2 h at room temperature with 10 µg of the antibody, followed by the 3 washes with PBS to get rid of the unbound antibodies. The rb79rich protocol required a longer incubation time and a larger bead volume (100 µL) coupled to rb79. Antibody-Agarose-beads-protein A/G complexes were incubated overnight at +4 °C and washed the next day 3 times with the ice-cold PBS followed by 100 µL of 2× a sample buffer containing: 125 mM Tris-HCl, pH 6.8, 4% SDS, 5% ß-mercaptoethanol, 20% glycerol, 0.01% Bromophenol Blue, and then boiled for 5 min at 95 °C prior to the SDS-PAGE procedure described above.

### 4.9. FACS

Flow cytometry assays were performed on cell populations cultured in triplicate in 25 cm^2^ flasks as originally described [19]. Approximately 106 cells were fixed with 1 mL of ice-cold 70% methanol for 30 min. After fixing, cells were pelleted by centrifugation to remove the fixatives, washed three times with phosphate-buffered saline (PBS) at 4 °C, resuspended in 100 μL PBS, treated with 100 μL RNAse A (1 mg/mL, Sigma, Saint-Quentin-Fallavier, France), and stained with rb79 at 50 μg/mL. The stained cells were stored at 4 °C in the dark and analyzed within 2 h. The stained samples were measured on a FACScan flow cytometer (Becton–Dickinson, San Jose, CA, USA). Data were acquired for at least 7000 events with a variation coefficient of less than 5%, and green fluorescence was measured using a fluorescence detector 3 (FL3) on the x-axis. The data were stored and analyzed using CellQuest software (Version 5.1).

### 4.10. Immunohistochemistry

Paraffinized human prostate anonymous tissue sections from 21 prostatectomies were obtained from the Department of Cell Pathology, Hôpital St. Vincent de Lille. Paraffin-embedded prostate tissue sections were subjected to conventional deparaffinization followed by antigen retrieval using citrate buffer at 95 °C in a water bath. After saturation in the solution containing 1% BSA and 0.05% Triton X100 in PBS-gelatin, the prostate sections were incubated with the specific antibodies, such as rabbit polyclonal anti-TRPV6 antibodies (rb79–82, at 1/200 of the initial concentration of 1 µg/µL), overnight at 4 °C (Table 4). Donkey polyclonal anti-rabbit and anti-mouse peroxidase-conjugated secondary antibodies (Chemicon International, Temecula, CA, USA; 1/200) were used. After revelation with diaminobenzidine (Sigma-Aldrich, Saint-Quentin-Fallavier, France), images were analyzed using a Zeiss Axioscope microscope (Carl Zeiss, Zaventem, Belgium) and Leica Image Manager software (Leica Geosystems AG Heinrich, Heerbrugg, Switzerland). Details on antibodies, dilutions, and staining characteristics carried out in the Department of Cell Pathology are given in Table 4. Immunohistochemistry was performed automatically using a Benchmark XT automated slides stainer (Ventana Medical Systems, Inc., Tucson, AZ, USA) following established protocols, and detection was performed using an IVIEW-DAB detection system (N760-500, Ventana Medical Systems, Inc., Oro Valley, AZ, USA).

### 4.11. Plasmids

The whole TRPV6 cDNA containing the 5′-UTR on the pCAGGS vector was provided by Dr. Ulrich Wissenbach from the Universität des Saarlandes, Germany. This sequence was used to obtain a final vEF1ap-5′UTR-TRPV6_CMVp-mCherry vector (E-Zyvec, Loos, France), which was nucleofected into the cells, and the transfection rate was evaluated using a control vEF1ap-5′UTR_CMVp-mCherry vector. pTRPV6-eYFP and pOrai1-YFP vectors were used as previously described [12].

### 4.12. HAP-1^trpv6−/−^ Model Creation

The HAP-1^*trpv6−/−*^ cell model has been created using the HAP-1 cell line, which is a near-haploid human cell line that was derived from the male chronic myelogenous leukemia (CML) cell line KBM-7 [22]. A knockout model of the HAP-1 cell line has been created by Horizon Discovery Ltd., Cambridge, UK, using CRISPR/CAS-9 technology and gRNA (CTCGCACCAGGTTCATGTTC) targeting exon 4 and yielding a 1 bp insertion in this exon (Supplementary Figure S1). This insertion resulted in the reading frame shift and a premature stop codon. The C insertion has been validated by the sequencing reaction using the sequencing primer CTACCCCTGAGGGAAAGAGACTG.

### 4.13. Antibody Production

The 15 amino acid antigens were synthesized, coupled to a KLH protein at its N-terminus, and injected into the rabbits once per week for four weeks following the final bleed (Eurogentec, Ltd., Seraing, Belgium). The serum was tested by ELISA using antigen-coated plates, followed by affinity purification in columns against the same bound antigen. Final affinity-purified antibodies were obtained and diluted 50/50 *v*/*v* with glycerol and stored at −20 °C.

### 4.14. Reagents

All reagents were purchased from Sigma (Sigma, L’Isle d’Abeau Chesnes, France) unless otherwise specified.

### 4.15. Data Analysis

For each type of experiment where the data were analyzed using statistics, the data were accumulated from at least three independent experiments. Data were analyzed using Origin 7.0 (MicroCal Software Inc., Northampton, MA, USA) software. Results were expressed as Mean ± S.E.M., where appropriate. N equals the number of series of experiments and n equals the number of cells used in the study. ANOVA was used for the statistical comparison of the differences, and *p* < 0.05 was considered significant. In the graphs, (*) and (**) denote statistically significant differences with *p* < 0.05 and *p* < 0.01, respectively.

## 5. Patents

This work is an integral part of patent EPO, number EP21306438. Title: “ANTIBODIES AGAINST EXTRACELLULAR EPITOPES OF HUMAN TRPV6 CHANNEL AND THEIR DIAGNOSTIC AND THERAPEUTIC USES”, inventors: Dr. V’yacheslav Lehen’kyi, Dr. Aurélien Haustrate, and Prof. Natalia Prevarskaya, filed on 14 October 2021. (Submission number 1000504057; Application number EP21306438.9; No. to be used for priority declarations EP21306438; Date of receipt: 14 October 2021).

## Figures and Tables

**Table 1:** Primers and siRNA (in italics, primers for qPCR).

Accession Number	Forward	Backward	Expected Size (b.p)
TRPV6, NM_018646	CCCTCAGTGTCTCGAAGTAC	TCAGATCTGATATTCCCAGCTC	134
*TRPV6, NM_018646*	*CCCAAGGAGAAAGGGCTAAT*	*TTGGCAGCTAGAAGGAGAGG*	145
TRPV5, NM_019841	TCTTCCAACTTCCTCCCTG	CCTCACTAAGGTTCAGTCCAAG	115
ACTB, NM_001101	CAGAGCAAGAGAGGCATCCT	GTTGAAGGTCTCAAACATGATC	209
*HPRT, NM_000194*	*GGCGTCGTGATTAGTGATGAT*	*CGAGCAAGACGTTCAGTCCT*	134
TRPV6 siRNA-1	5′-CCUGCUGCAGCAGAAGAGG (dTdT)-3′		
TRPV6 siRNA-2	5′-GACUCUCUAUGACCUCACA (dTdT)-3′		
TRPV6 siRNA-3	5′-CGUCAUGUACUUCGCCCGA (dTdT)-3′		
TRPV6 siRNA-4	5′-CCUCCUCAUUGCCAUGAUG (dTdT)-3′		
siLuciferase, AB_490793	5′-CUUACGCCUGAGUACUUCGA (dTdT)-3′		

**Table 2:** Primers for TRPV6 mRNA profiling.

Target and Accession Number	Sequence
TRPV6, NM_018646	V6-ex1-F-1: AAGGCAGGAGACAGGAGAC
TRPV6, NM_018646	V6-ex1-F0: GACCTCTACAGGGAGACGG
TRPV6, NM_018646	V6-ex3-B2: CATAGAGCTCAGATGTCATGG
TRPV6, NM_018646	V6-ex4-F3: CAGAACATGAACCTGGTGC
TRPV6, NM_018646	V6-ex5-B3: CGATCTCCTCACTGTTCACA
TRPV6, NM_018646	V6-ex6-B4: CTGTCGTAGGACAGCAACAG
TRPV6, NM_018646	V6-ex7-B5: GGTGGTGATGATAAGTTCCAG
TRPV6, NM_018646	V6-ex8-F4: GGTGCCATATATCTGCTGTAC
TRPV6, NM_018646	V6-ex9-B6: CTACCAGCAGGATGATGATAG
TRPV6, NM_018646	V6-ex10-B7: GGATGGTCTGTCCAAAGAAG
TRPV6, NM_018646	V6-ex11-Fsq: GGCTGGTGCAACGTCATGTAC
TRPV6, NM_018646	V6-ex12-Fex: ATTCTGCTGGCTGATGGC
TRPV6, NM_018646	V6-ex13-Bex: CGATGATGGTAAGGAACAGC
TRPV6, NM_018646	V6-ex14-F5: TTGTGGCCACCACGGTG
TRPV6, NM_018646	V6-ex15-B8: AGGTACTTCGAGACACTGAGG

**Table 3:** List of the primers used for RNA-profiling and the expected sizes of the PCR amplicons.

Primers	Expected Size, bp	Targeted Exon
F-1/B2	480	(5′-UTR)-2
F0/B2	460	(5′-UTR)-2
F1/B1	150	1-2
F1/B2	330	1-2-3
F3/B3	150	4-5
F3/B4	290	4-5-6
F3/B5	500	4-5-6-7
F4/B6	210	8-9
F4/B7	260	8-9-10
Fsq/Bex	240	11-12
Fex/Bex	150	12-13
F5/B8	280	14-15

**Table 4:** List of antibodies, their epitopes, and conditions of use in the case of WB and/or IHC.

Antibody	Design/Manufacturer	Clone	Epitope	Dilution	IHC Pretreatment and Incubation
TRPV6	Design: Dr. LEHEN’KYI, Produced: EUROGENTEC, Ltd., Seraing Belgium	rb79	QEAYMTPKDDIRLVG	1/200 IHC	CC2 STD 32 min
TRPV6	Design: Dr. LEHEN’KYI, Produced: EUROGENTEC, Ltd., Seraing, Belgium	rb80	QRRESWAQSRDEQNL	1/500 WB	
TRPV6	Design: Dr. LEHEN’KYI, Produced: EUROGENTEC, Ltd., Seraing, Belgium	rb81	HTRGSEDLDKDSVEKL	1/500 WB	
TRPV6	Design: Dr. LEHEN’KYI, Produced: EUROGENTEC, Ltd., Seraing, Belgium	rb82	TEDPEELGHFYDYPMA	1/500 WB	

## Supplementary Materials

The following supporting information can be downloaded at: https://www.mdpi.com/article/10.3390/ijms24010419/s1.
