# Supplementary figures and images for: A Novel Anti-TRPV6 Antibody and Its Application in Cancer Diagnosis In Vitro
Source: Int J Mol Sci. 2022 Dec 27;24(1):419. doi: 10.3390/ijms24010419 (PMC9820453; doi:10.3390/ijms24010419)

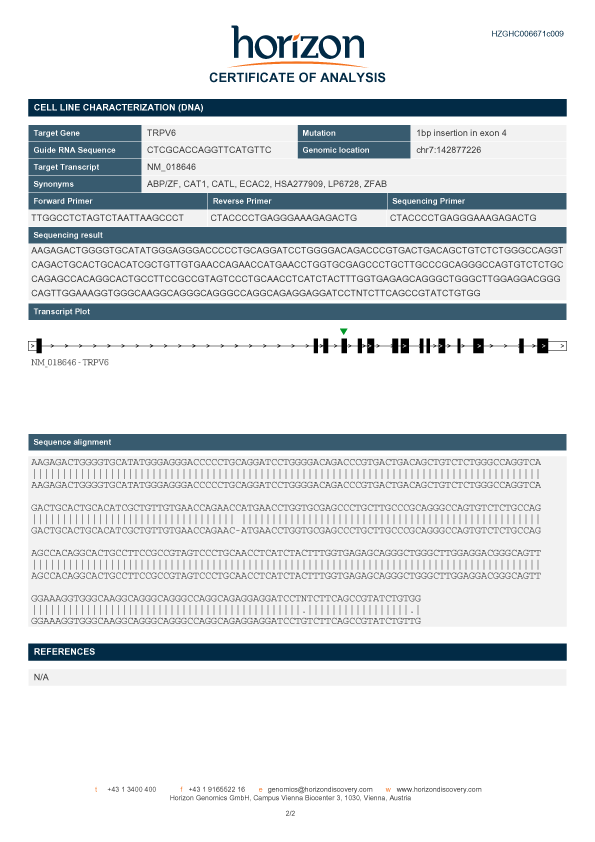

Supplement: Supplementary file 1 [file ijms-24-00419-s001.zip › Figure S1.tif]

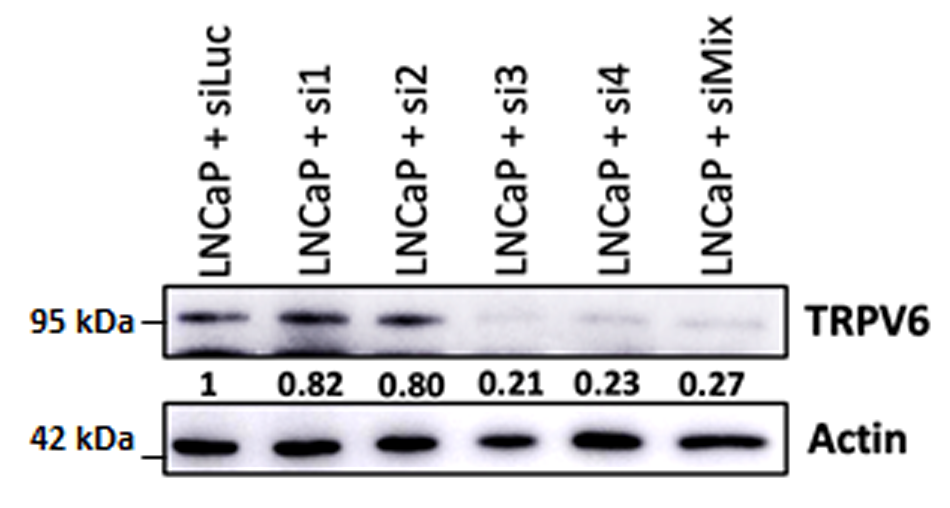

Supplement: Supplementary file 1 [file ijms-24-00419-s001.zip › Figure S2.tif]
